# The Effects of Radiotherapy on Microhardness and Mineral Composition of Tooth Structures

**DOI:** 10.1055/s-0042-1746414

**Published:** 2022-08-09

**Authors:** Patcharawat Siripamitdul, Pimduean Sivavong, Thanaphum Osathanon, Chawalid Pianmee, Wiroj Sangsawatpong, Chalermchart Bunsong, Dusit Nantanapiboon

**Affiliations:** 1 Department of Operative Dentistry, Faculty of Dentistry, Chulalongkorn University, Bangkok, Thailand; 2 Dental Stem Cell Biology Research Unit and Department of Anatomy, Faculty of Dentistry, Chulalongkorn University, Bangkok, Thailand; 3 Dental Department, Surin Hospital, Surin, Thailand; 4 Dental Department, Chonburi Cancer Hospital, Chonburi, Thailand; 5 Department of Radiation Oncology, Chonburi Cancer Hospital, Chonburi, Thailand; 6 Dental Material Research and Development Center, Faculty of Dentistry, Chulalongkorn University, Bangkok, Thailand

**Keywords:** radiotherapy, tooth structure, microhardness, SEM-EDS, FT-Raman

## Abstract

**Objective** The purpose of this study was to evaluate the microhardness and mineral composition alterations in enamel and dentine after radiotherapy.

**Materials and Methods** Forty human maxillary premolar teeth (20 pairs) were assigned to nonirradiated and irradiated groups, the latter irradiated by fractional radiation to achieve a total dose of 70 Gy. Microhardness measurement was performed on a Knoop microhardness tester. Chemical components were analyzed using energy dispersive spectroscopy and Fourier transform Raman spectroscopy. The morphology was observed using a scanning electron microscope. The microhardness data were analyzed using a paired *t* -tested and one-way repeated analysis of variance (ANOVA), and the mineral composition data using related-samples Wilcoxon signed rank test and related-samples Friedman's two-way ANOVA by ranks.

**Results** The irradiated teeth had a significantly lower microhardness in both enamel and dentine compared with the nonirradiated teeth. The irradiated dentine at 50 μm from the external tooth surface at the cemento-enamel junction showed the lowest microhardness compared with other locations. There was no statistically significant difference in calcium:phosphate ratio and chemical components. There was a reduction in protein:mineral ratio in dentine and at the cemento-enamel junction after irradiation. The irradiated teeth exhibited crack lines at the dentine-enamel junction and in dentine.

**Conclusion** Fractional radiation reduced microhardness in both enamel and dentine. The cervical dentine exhibited the highest microhardness reduction compared with other enamel and dentine locations.

## Introduction

Radiation caries is one of the common conditions affecting patients undergoing head and neck radiation therapy (HNRT). Approximately 25% of HNRT patients are diagnosed with radiation caries, 1 the onset being in the range of 3 to 12 months after the completion of radiotherapy. Progression is rapid and aggressive and patients' long-term quality of life is negatively affected. 2 Radiation caries most often occurs at locations which are generally not susceptible to dental caries, such as cusp tips, incisal edges, and lower anterior teeth, 2 and the underlying dentine appears as a brown/blackish discoloration. As untreated lesions progress, a fracture of the cusp tip or incisal edge occurs. One of the most common features of radiation caries is widespread cervical lesions, leading to the delamination of enamel and damage to the underlying dentine, eventually leading to loss of the crown. 3

However, the etiology of radiation caries is unclear. Two possibilities are the direct effects of ionizing radiation on the tooth structure and the indirect effects on the surrounding structures such as salivary glands and oral soft tissues. 4 The clinical feature of radiation caries differs from bacterial carious lesion. Radiation caries occur at the location that is generally not usually susceptible to dental caries formation. Since the characteristics of radiation caries are different from nonradiation caries, one reason could be the direct effect of ionizing radiation on tooth structure. The results of previous studies on the mechanical and physical properties of both enamel and dentine after radiation are still controversial. 5 6 7 8 9 10 11 Some studies found the increase of mechanical properties while others found the decrease or no alteration. Different tooth types and the different oral microenvironment of each individual could have an effect on the different investigation's outcome.

The result of the previous study was unable to conclude the effect of the radiation on the tooth structure including the mechanical and chemical properties. Therefore, the present study aimed to evaluate the microhardness and mineral composition of enamel and dentine after radiotherapy using pair teeth from the same individual.

## Materials and Methods

### Teeth Collection

The study protocol was approved by the Human Research Ethics Committee of the Faculty of Dentistry, Chulalongkorn University, Bangkok, Thailand (HREC-DCU 2021-011). Forty (20 pairs, left and right) of sound human maxillary premolar teeth, extracted for orthodontic reasons, were collected from 20 patients, aged between 18 and 25 years. The teeth were similar in size and were without crack lines, carious lesions, restorations, fluorosis, or hypoplasia. The teeth were cleaned and disinfected in 0.1% thymol solution, stored in deionized water on a sieve tray at 4°C, and used within 3 months after extraction.

### Irradiation Procedure

The 20 right maxillary premolar teeth in the experimental group were removed from the deionized water, blotted with paper until dry, and exposed to fractional radiation(2 Gy/fraction/day, 5 days/week, for 7 weeks) to achieve a total dose of 70 Gy, using an intensity-modulated irradiator (intensity-modulated radiation therapy, Varian RapidArc, Varian Medical Systems, Palo Alto, California, United States) at Chonburi Cancer Hospital, Chonburi, Thailand. The radiation dose was calculated with a computerized tomography scan (Aquilion LB TSX-201A, Toshiba Corp., Tokyo, Japan) using the Eclipse program (Eclipse Veterinary Software Ltd., Great Chesterford, England) so that all specimens obtained an equal amount of radiation. After radiation, the sieve tray containing the experimental teeth was immersed in 20 mL deionized water at 37°C for 7 days.

The 20 left maxillary premolar teeth in the control group were similarly immersed in deionized water, changed daily in both groups.

### Specimen Preparation

A pair of maxillary premolar teeth from the same patient were aligned parallel in a silicone mold, 30 mm wide × 30 mm long × 30 mm deep, on the same plane so that the positions of microhardness and mineral composition measurements were as close as possible for each pair. Polyester resin was poured into the mold, allowed to set, and the teeth longitudinally sectioned into mesial and distal halves using a low speed cutting machine (Isomet 1000, Buehler Ltd., Lake Bluff, Illinois, United States) and the pulpal remnants removed.

### Microhardness Measurement

The mesial half was assigned for microhardness testing, and polished with 800-, 1,000-, and 1,200-grit silicon carbide paper and a flannel polishing head in combination with 0.05-μm alumina abrasive powder using a polishing machine (MINITECH 233, PRESI, Eybens, France). Specimens were cleaned with deionized water using an ultrasonic cleaner (Ultrasonic Cleaner 5210, Branson Ultrasonic Corp., Brookfield, Connecticut, United States) for 15 seconds.

**Fig. 1:**
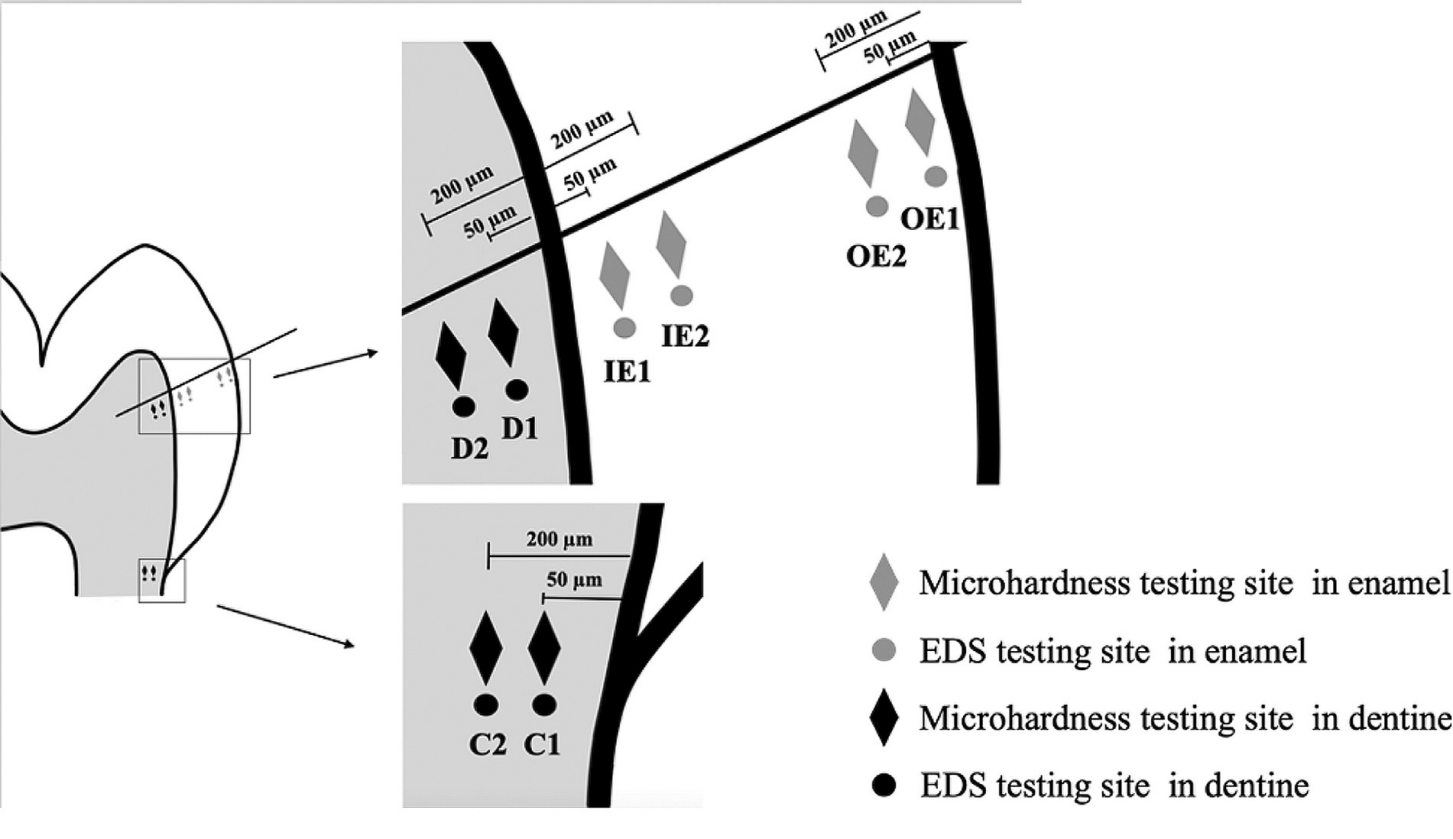
Measurement sites in enamel and dentine.

Microhardness measurements of enamel, dentine at the dentine-enamel junction, and dentine at the cemento-enamel junction were performed with a Knoop microhardness tester (FM810, Future-Tech Corp., Kanagawa, Japan) with a 50-g load applied for 15 seconds. The sites of microhardness measurement were determined by measuring occlusally from the cemento-enamel junction for 4 mm on the palatal side ( Fig. 1 ). At this level, a line was marked obliquely upward and perpendicular to the dentine-enamel junction, and the indentations performed below the marked line. For enamel, four indentations were performed at a distance of 50 and 200 μm from both the dentine-enamel junction (IE1, IE2) and the external tooth surface (OE1, OE2). In dentine, four indentations were performed at a distance of 50 and 200 μm from the dentine-enamel junction (D1, D2) and from the external tooth surface at the cemento-enamel junction (C1, C2).

The microhardness mean values of all specimens were calculated for comparation between irradiated and nonirradiated groups. The effect of irradiated enamel and dentine were also evaluated by the difference in microhardness at each location.

### Scanning Electron Microscopy and Energy Dispersive Spectroscopy Analysis

Five pairs of mesial half specimens were randomly selected for scanning electron microscopy and energy dispersive spectroscopy (SEM-EDS) analysis, coated with gold/palladium and examined by SEM (Hitachi Corp., Tokyo, Japan). A point analysis was performed underneath the marked line for microhardness measurement for eight sites per specimen at 15 kV ( Fig. 1 ). The mean calcium:phosphate ratios from each specimen were calculated for comparison between irradiated and nonirradiated specimens at each location.

### Fourier Transform Raman Spectroscopy

Three pairs of distal half specimens were randomly selected for Fourier transform (FT) Raman spectroscopy (HORIBA Jobin Yvon, Inc., Edison, New Jersey, United States) with a near-infrared (785 nm) laser. The spectrum data were collected over the range of 400 to 3700 cm ^−1^ . Three sites of measurement were determined by measuring occlusally from the cemento-enamel junction for 4 mm on the palatal side. At this level, a line was marked obliquely upward and perpendicular to the dentine-enamel junction, and the indentations performed below the marked line. For enamel and dentine, the indentations were performed at a distance of 2 mm from the dentine-enamel junction. For cemento-enamel junction, the indentations were performed at a distance of 2 mm from the external tooth surface at the cemento-enamel junction level.

The peaks at 2931 and 960 cm ^−1^ of Raman spectroscopy represent the C-H stretch of protein and the stretching mode of the phosphate (PO _4_ ) group, respectively. 12 The mean protein:mineral ratio was compared between the irradiated and nonirradiated groups in each region by calculating the area under the graph using SigmaPlot (Systat Software Inc., San Jose, California, United States).

### Statistical Analysis

The statistical analysis was performed using SPSS version 26.0 (IBM, Armonk, New York, United States). A statistically significant difference was set at *p* < 0.05. The microhardness data were analyzed using paired *t* -tests and one-way repeated analysis of variance (ANOVA). The mineral composition data were analyzed using related-samples Wilcoxon signed rank test and related-samples Friedman's two-way ANOVA by ranks. Graphs were generated using Prism 8 (GraphPad Software, San Diego, California, United States).

## Result

### Microhardness Examination

**Fig. 2:**
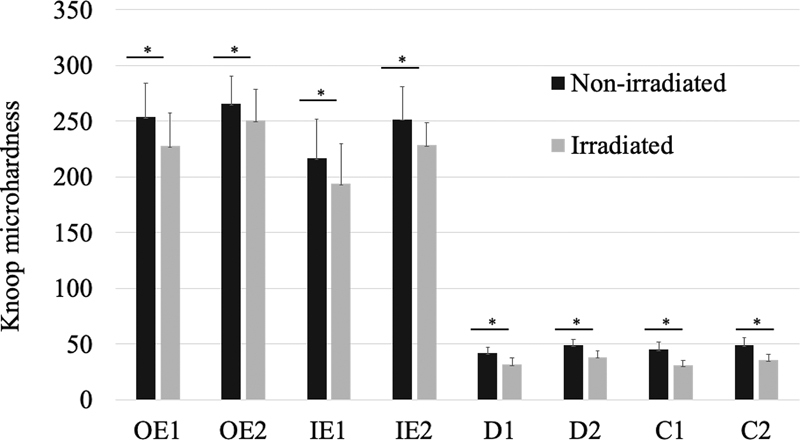
Mean Knoop microhardness of nonirradiated and irradiated tissue at each site. Asterisks (*) indicate significant differences ( *p* < 0.05). Vertical bars indicate standard deviations.

**Fig. 3:**
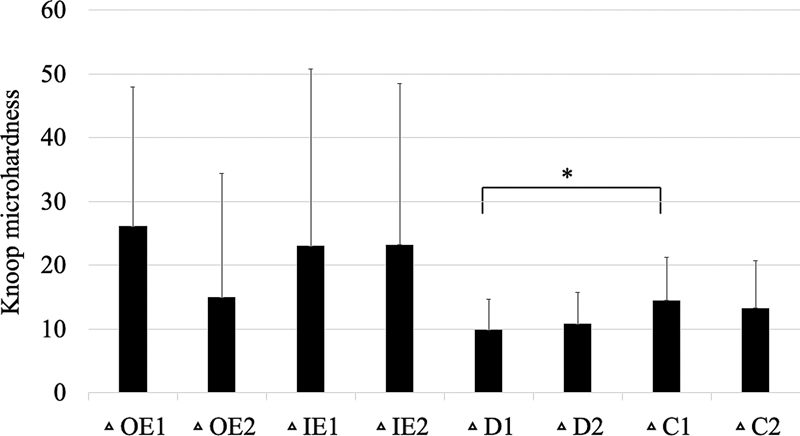
The mean differences in Knoop microhardness between irradiated teeth to nonirradiated teeth at the measurement sites in enamel and dentine. * indicates a statistically significant difference ( *p* < 0.05). Vertical bars indicate standard deviations.

The irradiated enamel had a significantly lower microhardness at all locations (OE1, OE2, IE1, IE2) compared with the nonirradiated control ( Table 1 and Fig. 2 ). The mean differences in microhardness values (delta; Δ) between nonirradiated and irradiated enamel were 26.1 ± 21.9, 14.9 ± 19.4, 23.0. ± 27.7, and 23.2 ± 25.3 at OE1, OE2, IE1, and IE2 locations, respectively ( Fig. 3 ). When comparing the delta microhardness values among all locations of enamel, the ΔOE1, ΔOE2, ΔIE1, and ΔIE2 were not significantly different.

**Table 1:** Mean Knoop microhardness of nonirradiated and irradiated tissue at each site

Locations	Knoop microhardness (mean ± SD)		
	Nonirradiated	Irradiated	*p* -Value
OE1	253.62 ± 30.36	227.54 ± 30.11	0.000
OE2	265.45 ± 24.66	250.51 ± 27.85	0.003
IE1	216.88 ± 34.87	193.89 ± 35.77	0.002
IE2	251.73 ± 28.86	228.55 ± 20.38	0.001
D1	41.80 ± 5.27	31.96 ± 5.53	0.000
D2	49.19 ± 5.03	38.42 ± 5.53	0.000
C1	45.16 ± 6.37	30.67 ± 4.40	0.000
C2	49.08 ± 6.59	35.81 ± 5.16	0.000

Abbreviation: SD, standard deviation.

Note: *p* -Value less than 0.05 is statistically significant.

For dentine, the microhardness of irradiated dentine at all locations (D1, D2, C1, C2) was significantly lower than the nonirradiated control ( Table 1 and Fig. 2 ). The mean differences in microhardness values (delta; Δ) between nonirradiated and irradiated dentine were 9.8 ± 4.9, 10.8 ± 4.96, 14.5 ± 6.7, and 13.3 ± 7.5 at D1, D2, C1, and C2 locations, respectively. When comparing the delta microhardness values among all locations of dentine, the ΔC1 was significantly greater than the ΔD1 ( Fig. 3 ). However, Δ values among the other locations were not significantly different.

### Chemical Composition

The calcium and phosphate components of dentine and enamel were found to be in a similar ratio in both the nonirradiated and irradiated teeth ( Table 2 ), thus the radiation did not significantly alter the calcium:phosphate ratio at any of the measurement sites, compared with the nonirradiated group.

**Table 2:** Calcium:phosphate ratio in nonirradiated and irradiated in enamel and dentine

Locations	Ca:P ratio (mean ± SD)	
	Nonirradiated	Irradiated
OE1	2.23 ± 0.03	2.25 ± 0.02
OE2	2.23 ± 0.03	2.25 ± 0.02
IE1	2.23 ± 0.06	2.25 ± 0.04
IE2	2.22 ± 0.05	2.25 ± 0.03
D1	2.24 ± 0.08	2.22 ± 0.03
D2	2.23 ± 0.04	2.22 ± 0.02
C1	2.25 ± 0.05	2.23 ± 0.05
C2	2.28 ± 0.03	2.26 ± 0.04

Abbreviations: Ca:P ratio, calcium:phosphate ratio; SD, standard deviation.

**Fig. 4:**
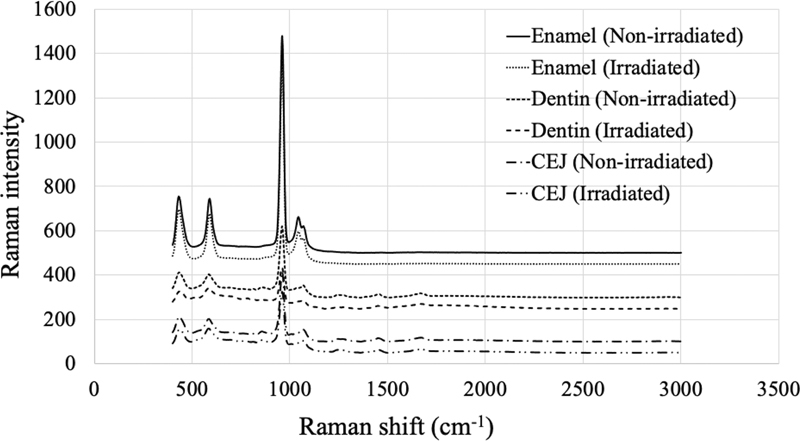
Raman spectral analysis of enamel, dentine, and cemento-enamel junction in irradiated and nonirradiated teeth.

Representative Raman spectra for enamel, dentine, and cemento-enamel junction of the nonirradiated and irradiated groups are presented in Fig. 4 . The protein-to-mineral ratio of dentine and cemento-enamel junction was decreased after radiation. However, the enamel part could not be interpreted since the protein-to-mineral ratio in enamel approached zero ( Table 3 ).

**Table 3:** Protein:mineral ratio

Subject	Protein-to-mineral ratio					
	Enamel		Dentine		CEJ	
	Nonirradiated	Irradiated	Nonirradiated	Irradiated	Nonirradiated	Irradiated
1	0.00	0.00	0.017	0.012	0.024	0.009
2	0.00	0.00	0.029	0.020	0.022	0.016
3	0.00	0.00	0.022	0.017	0.022	0.012

Abbreviation: CEJ, cemento-enamel junction.

### Scanning Electron Microscopy and Energy Dispersive Spectroscopy Analysis

**Fig. 5:**
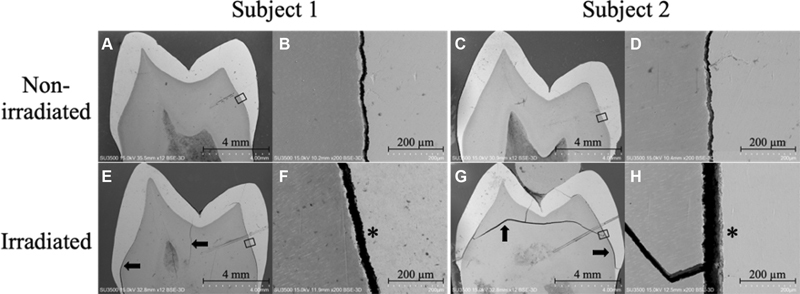
Representative scanning electron microscopy (SEM) images illustrating the tooth structure in the nonirradiated and irradiated groups. ( **A** , **C** , **E** , **G** ) 12× magnification. ( **B** , **D** , **F** , **H** ) Area within the square of A, C, E, and G, respectively, at 200× magnification. ( **A** , **C** ) Comparison of a nonirradiated tooth with an irradiated tooth ( **E** , **G** ). The crack line (arrowed) is present in the dentine and at the dentine-enamel junction of irradiated tooth. ( **B** , **D** ) Comparison of a nonirradiated with an irradiated tooth ( **F** , **H** ), at the dentine-enamel junction site. The crack line at the dentine-enamel junction in the irradiated tooth is wider than that in the nonirradiated tooth (asterisks).

Representative scanning electron micrographs of the enamel and dentine of nonirradiated and irradiated teeth from two subjects are shown in Fig. 5 . Irradiated specimens exhibited more crack lines than the nonirradiated specimens and were wider in dentine. Cracks at the dentine-enamel junction of the irradiated specimens were wider than those in the nonirradiated specimens.

## Discussion

The present study described the microhardness and mineral composition in enamel and dentine of teeth exposed to radiation. In order to control the biological difference among individuals, we assigned pair samples of left and right maxillary premolar teeth from each patient to nonirradiated and irradiated groups. We also utilized the radiation exposure methods in the way that the whole teeth were subjected to fractional radiation using the intensity-modulated irradiator mimicking the clinical situation of radiotherapy in head and neck cancer patients. 13

One experimental parameter that should be first considered in order to understand the interpretation is the storage medium. The storage medium indeed affects the physical parameter of tooth structures. However, the deionized water was employed in the present study similar to other previous studies. 14 Our rationale was that the radiation could damage the salivary gland. In the case of exposure to the high doses of radiation, the salivary glands were permanently destroyed. The stimulated whole saliva was dramatically reduced. The unstimulated and stimulated parotid saliva was reduced to 20% while the other salivary glands were reduced to 50% after 2 weeks. 15 The alteration of electrolyte in saliva composition during and after radiotherapy of the head and neck region was the reduction of bicarbonate and phosphate which played an important role in buffer capacity. 15 Therefore, the deionized water was considered to simulate the worst-case scenario in the irradiated patient without saliva remineralization.

The present study showed that microhardness of both enamel and dentine was significantly less in irradiated teeth compared with nonirradiated teeth, which is consistent with other publications. 5 6 9 16 On the contrary, there are reports that there was no statistically significant difference of microhardness of teeth after irradiation. 10 11 de Barros da Cunha et al 11 found that a single-dose irradiation only slightly lowered the microhardness of the cervical enamel and there was no alteration in the microhardness in other areas. However, others reported that the use of fractional radiation significantly lowered the microhardness in enamel and dentin. 5 6

The lower microhardness in irradiated teeth could be due to the decarboxylation process initiated by radiation, causing the elimination of carboxyl groups. 16 Radiation acts at the junction of the hydroxyapatite crystal and the collagen fibers, and at the calcium atom between the protein side chain carboxylate groups and apatite phosphate groups. As a result, bidentate complexes with phosphate groups are formed, and the calcium atom links to the complex instead of collagen fibers to the hydroxyapatite crystal. This consequently results in a poor attachment between the hydroxyapatite crystals and collagen fibers. The FT-infrared microscopy results illustrate the instability of the phosphate group in a hydroxyapatite crystal after irradiation. Furthermore, carbon dioxide, which is a by-product of decarboxylation, could induce microcracks in the tooth structure. 16

Another mechanism of the reduction in the microhardness in irradiated teeth could be a consequence of an oxidation reaction. Radiation breaks down the water molecules in tooth structure, resulting in the release of the reactive oxygen species 10 which can affect the proteolysis of collagenous and noncollagenous protein. 17 It has also been found that radiation can activate a matrix metalloproteinase enzyme, MMP-20, which can break down collagen type IV, especially at the inner enamel and dentine-enamel junction. 18 This indirect effect of MMP-20 can lead to an increase in protein destruction and hence the lower physical properties of enamel and dentine. 18

The result from the present study showed that the irradiated teeth had significantly lower microhardness at all enamel and dentine locations compared with the nonirradiated control. The comparison of the mean delta microhardness of each location in the enamel region was not statistically different. The explanation for this observation is that enamel has a high amount (approximately 96%) of the inorganic component, mainly hydroxyapatite, and has only a small amount of organic component, mainly the organic matrix of protein and water. 19 Hence, there is less of an effect of the oxidation reaction of water molecules on enamel compared to dentine. For dentine, after irradiation, the difference between the microhardness at the cemento-enamel junction was greater than that at the dentine-enamel junction region. This could be because the amount and diameter of the dentinal tubules at the cemento-enamel junction level are greater and wider, respectively, compared to those at the dentine-enamel junction, 20 resulting in more dentine tubular fluid in tubules at the cemento-enamel junction. Hence, the oxidative reaction of water could affect dentine microhardness at the dentine around the cemento-enamel junction, more so than at the dentine-enamel junction.

Considering the mineral composition after irradiation, the result showed that the calcium:phosphate ratio in both enamel and dentine were not significantly different. Similarly, previous reports have also shown that there was no difference in calcium:phosphate ratio in teeth irradiated by 20, 40, and 70 Gy, 11 implying that radiation did not affect the amount of calcium and phosphate in the hydroxyapatite crystal.

Representative Raman intensity peaks at 430 and 960 cm ^−1^ of phosphate between the nonirradiated and irradiated teeth were not different, indicating that there was no obvious alteration in phosphate crystals. The result corresponds to the EDS values that showed no alteration in the mineral structure. When the protein-to-mineral ratio was taken into account, there was a lower value in dentine and at the cemento-enamel junction region, which could be explained by the proteolytic destruction of the organic component in irradiated teeth as described above. The results of the present study were consistent with the study performed by Lu et al 9 who reported no difference in FT-Raman peaks between the nonirradiated and irradiated teeth, and also showed the reduction of the protein-to-mineral ratio in dentine. However, in the present study the result in the enamel could not be interpreted since the protein-to-mineral ratio in enamel approached zero, which might be the consequence of the small amount of protein in enamel.

As shown in the SEMs, the irradiated teeth exhibited more crack lines than the nonirradiated teeth, which was related to the effect on microhardness. The cracks were more obvious in dentine, and this could be due to the relatively lower compressive strength of dentine compared with the enamel. 21 The width of the crack at the dentine-enamel junction was wider in the irradiated specimens, which showed that there was more damage to the irradiated specimens.

The present study found an alteration in the microhardness and in the reduction of the protein-to-mineral ratio after radiotherapy, and it can therefore be inferred that there are direct effects of the radiation on tooth structure which could be one of the factors contributing to the occurrence of radiation caries. Radiation caries tends to result in extensive lesions at cervical areas, which is consistent with the result of this present study. Moreover, an indirect effect of radiation caries is the progression of lesions due to plaque retention at the cervical area, hyposalivation, trismus, and oral mucositis which are side effects after radiotherapy. 4 Therefore, further studies should seek a method of preventing or decreasing the side effects of radiotherapy on the cervical dentine.

A limitation of the present study is that the experiment was conducted using the maximum radiation dose for head and neck cancer therapy, being 70 Gy. 13 In fact, patients may be exposed to a lower radiation dose, possibly causing different effects from those obtained in this study, so more information is required on the amount of radiation dose to the tooth. Moreover, the study also measured the immediate effects on tooth structure after radiation, thus any delayed changes induced by radiation should be investigated. In this regard, an ageing process such as pH cycling could have been performed in order to simulate postradiation caries. Lastly, further study with randomized sampling could be employed to reduce the possible bias.

## Conclusion

Fractional radiotherapy of 70 Gy results in a lower microhardness of both enamel and dentine. Dentine in the cervical region exhibits lower microhardness compared with dentine adjacent to the enamel in the irradiated teeth. The irradiated dentine at 50 μm from the external tooth surface at the cemento-enamel junction level showed the lowest microhardness. However, the mineral composition of neither enamel nor dentine was affected by radiotherapy.
